# Supplementary material for: Conscious observational behavior in recognizing landmarks in facial expressions
Source: PLoS One. 2023 Oct 4;18(10):e0291735. doi: 10.1371/journal.pone.0291735 (PMC10550163; doi:10.1371/journal.pone.0291735)
Supplement: Attachment — Submitted filename: Response to Reviewers.pdf [file pone.0291735.s001.pdf]

Response to Reviewers

Reviewer #1: The manuscript is described in a technically sound way. The scientific research with data that supports the conclusions is provided. Experiments have been conducted which is described in a elaborate way which is also provided with appropriate results. Care has to be taken as there are repetition of words and sentences.

Thank you very much for your valuable suggestion. We re-read our paper and realized that there were several sentences that were not needed or ambiguous. We have double-checked the content and corrected instances of word and sentence repetition to make our paper better.

Reviewer #2: Constructed Bayesian models to analyze influence of personality traits and observational behaviors on expressional judgments. The objective of the work is met to reasonable extent.

1) what threshold of the parameter is deciding upon the minimum or maximum facial expressions in situations.

[Line 119 - 120] We have added content about the criteria for determining the visible range. The size of the visible range was determined based on the content of the previous studies [line 136 – 138].

[Line 406 - 409 ] We have added a discussion of the effect of visible range size.

2) "The existence of a mechanism whereby personality traits lead to different conscious observational behaviors, and expressional judgments are based on information obtained through those observational behaviors." , little elaboration of the Mechanism is needed

[line 391 - 394] we add a few sentences to elaborate and discuss the Mechanism.

3) After deleting all identifiable information of the 79 participants and are storing the data, what is the actual data information size. Any particular reason for considering the data where female are 74 and male are 12 as participants. what is the impact of this uneven consideration of the data in terms of gender to the research carried.

[line 73 - 81] our participant sample size was 41(12 male and 29 female). As

you pointed out, the ratio of male to female participants is somewhat unbalanced. However, since we did not examine the effect of gender in this experiment, we do not believe that this imbalance would affect our main results. Our data showed a significant correlation between gender and personality traits, and using both variables simultaneously in the analysis may lead to multicollinearity. Thus, we left the “gender effect’ from our analyses. In the future, we would like to consider the gender effect in our research.

4)few more comparison on choosing the Bayesian model for the analysis. further the six expressions along with personality traits are graphed well. More insight on the assessment on personality traits can be added.
[Line 251 - 266] we add other results about happiness, sadness and surprise.
[Line 296 - 310] we add other results about happiness.

5) More information on personality trait score can be added.
[Line 124 - 128] we add new content to explain the TIPI-J and Big Five (by using a footnote).

6) Overall good analysis... more information to be added on the impact of adding more data than what is considered. how will the Bayesian model handle its efficiency with more number of participants.
[Line 157 -164] we add new content to talk about the Bayesian model.
